# An Explorative Study of Suicidal Risk in Women with Gynaecological Oncology Compared to Infertility

**DOI:** 10.1192/bjo.2026.11248

**Published:** 2026-06-30

**Authors:** Baidyanath Ghosh Dastidar, Sudarsan Ghosh Dastidar

**Affiliations:** 1 Calcutta National Medical College, Kolkata, India; 2 Ghosh Dastidar Institution for Fertility Research, Kolkata, India

## Abstract

**Aims:**

Women with infertility and those diagnosed with gynaecological malignancies experience significant psychological distress. While infertility-related suicidality has been increasingly studied, suicidal risk among women with gynaecological oncology remains underexplored, particularly in low- and middle-income settings. This study aimed to compare depression, anxiety, quality of life, and suicidal ideation between women with gynaecological oncology and women with general infertility.

**Methods:**

Women with infertility and those diagnosed with gynaecological malignancies experience significant psychological distress. While infertility-related suicidality has been increasingly studied, suicidal risk among women with gynaecological oncology remainsunderexplored, particularly in low- and middle-income settings. This study aimed to compare depression, anxiety, quality of life, and suicidal ideation between women with gynaecological oncology and women with general infertility.

**Results:**

Women with gynaecological oncology demonstrated significantly higher levels of depressive symptoms and anxiety compared to women with infertility, as measured by BDI and BAI scores (p <0.001). The oncology group also showed significantly greater global psychopathology on the SCL-90 Global Severity Index. Quality of life scores across physical functioning, emotional wellbeing, social functioning, and role limitations were markedly lower in the oncology group on the SF-36. Suicidal ideation was significantly more prevalent among women with gynaecological oncology compared to infertile women (p <0.01), indicating a higher level of suicidal risk in the oncology population.

**Conclusion:**

Women with gynaecological oncology demonstrated significantly higher levels of depressive symptoms and anxiety compared to women with infertility, as measured by BDI and BAI scores (p <0.001). The oncology group also showed significantly greater global psychopathology on the SCL-90 Global Severity Index. Quality of life scores across physical functioning, emotional wellbeing, social functioning, and role limitations were markedly lower in the oncology group on the SF-36. Suicidal ideation was significantly more prevalent among women with gynaecological oncology compared to infertile women (p <0.01), indicating a higher level of suicidal risk in the oncology population.

